# Fertility Dynamics and Life History Tactics Vary by Socioeconomic Position in a Transitioning Cohort of Postreproductive Chilean Women

**DOI:** 10.1007/s12110-022-09425-z

**Published:** 2022-05-25

**Authors:** Pablo José Varas Enríquez, Luseadra McKerracher, Nicolás Montalva Rivera

**Affiliations:** 1 Department of Human Behaviour, Ecology, and Culture, Max Planck Institute for Evolutionary Anthropology, Leipzig, Germany; 2 BirthRites Independent Research Group, Max Planck Institute for Evolutionary Anthropology, Leipzig, Germany; 3 Institute for Biodiversity and Ecosystem Dynamics, University of Amsterdam, Amsterdam, The Netherlands; 4 Department of Anthropology, Faculty of Social Sciences, Universidad de Chile, Santiago, Chile; 5 Department of Public Health and Aarhus Institute for Advanced Studies, Aarhus University, Aarhus, Denmark; 6 Society and Health Research Centre, Faculty of Humanities, Universidad Mayor, Santiago, Chile; 7 School of Public Health, Faculty of Sciences, Universidad Mayor, Santiago, Chile; 8 Millennium Nucleus on Sociomedicine (Sociomed), Santiago, Chile

**Keywords:** Human reproduction, Life history theory, Fertility, Evolutionary demography, Demographic transition

## Abstract

Globally, mortality and fertility rates generally fall as resource abundance increases. This pattern represents an evolutionary paradox insofar as resource-rich ecological contexts can support higher numbers of offspring, a component of biological fitness. This paradox has not been resolved, in part because the relationships between fertility, life history strategies, reproductive behavior, and socioeconomic conditions are complex and cultural-historically contingent. We aim to understand how we might make sense of this paradox in the specific context of late-twentieth-century, mid–demographic transition Chile. We use distribution-specific generalized linear models to analyze associations between fertility-related life-history traits—number of offspring, ages at first and last reproduction, average interbirth interval, and average number of live births per reproductive span year—and socioeconomic position (SEP) using data from a cohort of 6,802 Chilean women born between 1961 and 1970. We show that Chilean women of higher SEP have shorter average interbirth intervals, more births per reproductive span year, later age at first reproduction, earlier ages at last reproduction, and, ultimately, fewer children than women of lower SEP. Chilean women of higher SEP consolidate childbearing over a relatively short time span in the middle of their reproductive careers, whereas women of lower SEP tend to reproduce over the entirety of their reproductive lifespans. These patterns may indicate that different SEP groups follow different pathways toward declining fertility during the demographic transition, reflecting different life-history trade-offs in the process.

Most human populations have recently undergone or are currently undergoing demographic transitions in which all-cause mortality rates decline steeply and then fertility rates decline slightly less steeply, resulting in larger population sizes and changes in population age structure (Caldwell et al., 2006; Coale, 1984; Davis, 1945). These demographic changes accompany increases in the resource-richness of the ecological contexts in which they are occurring, especially increases in the abundance of food and energy (Livi-Bacci, 2012; Myrskylä et al., 2009). This pattern, in contrast to earlier human demographic transitions (e.g., the Neolithic demographic transition; Bocquet-Appel 2011) and to most demographic changes in other animals, presents an evolutionary and historic puzzle because additional resources can be converted into *increased* fertility, which, other things being equal, should be expected to confer fitness benefits.

Researchers from evolutionary demography and biological anthropology have sought to address this puzzle using life-history theory (Borgerhoff Mulder, 1998; Sear et al., 2016). The life-history framework holds that amounts of time and energy available to an organism over its life course are finite, such that time and energy allocated to one purpose over the lifespan, such as growth or somatic maintenance, cannot be allocated to other purposes, such as reproduction (e.g., mate-finding, pregnancy, lactation). In other words, life-history theory implies time and energy trade-offs between different aspects of an organism’s life, and these trade-offs are generally expected to offer net fitness benefits (Stearns, 1977). The offspring quantity versus offspring quality trade-off is the most-studied life-history trade-off in research regarding the demographic transition (Kaplan, 1996). That is, under some ecological conditions, the most fitness-enhancing strategy for a family may be to consolidate investment of resources in a relatively small number of very-high-quality offspring (offspring with, for example, good health, prestige, earning potential). Under other conditions, particularly under resource-poor conditions in which external morbidity and mortality risks are high, the better strategy may be to invest a relatively small amount of time and energy in each of a much higher number of children, improving the chances that at least a few children will survive to reproductive age and have children of their own (Kaplan, 1996; Hill & Kaplan, 1999; Kaplan et al., 2003; Leslie & Winterhalder, 2002; Shenk et al., 2016). While this quantity-quality trade-off appears important, other trade-offs related to the reproductive timing and parent-offspring resource allocation, such as reproducing now versus later or investing in reproduction versus (extra)somatic maintenance, may also play significant roles in accounting for how demographic transitions unfold in different ecological (including political) contexts (Hill & Kaplan, 1999; Lawson & Borgerhoff Mulder, 2016).

## Three Classes of Complementary Models

Three major classes of models have been advanced to explain the mechanics of fertility decline during the most recent demographic transition: risk/mortality models, economic/investment models, and cultural transmission models (Shenk et al., 2013). Previous work suggests that we can expect slightly different factors (e.g., pathogen load/sanitation, wealth/income, education/knowledge, support network size, availability of a wide range of cultural models) to exert relatively strong or weak effects on fertility-related or life-history variables under each of the three classes of models (e.g., Coall et al., 2016; McAllister et al., 2016; Shenk et al., 2013, 2016). We view the classes of models, outlined below, as complementary to one another, with each likely to contribute to variation in human life-history tactics during demographic transitions in differing amounts, depending on the historical and ecological specifics of the context in which the transition is occurring. That said, we also suggest that each of the groups of models can offer slightly different—albeit not mutually exclusive—predictions regarding which life-history variables are to be impacted by ecological resource richness and in which ways.

Risk/mortality models explain the decline in fertility predominantly as a response to infant mortality rates plummeting when sanitation, nutrition, and health care improve. Therefore, parents—consciously or unconsciously—observe that children are becoming increasingly likely to survive to adulthood and reduce their number of births accordingly by delaying marriage/reproduction and spacing births more widely apart (Coale, 1984). Versions of these models directly informed by life-history theory suggest that physiological development and aging patterns as well as energy storage and mobilization patterns will mitigate this process such that low-risk, abundant ecological conditions will favor slow, high-investment-high-likelihood-of-return strategies (Cervellati & Sunde, 2005; Chisholm et al., 1993; Galor, 2011; Leslie & Winterhalder, 2002; Quinlan, 2007). Our view is that the risk/mortality framework predicts that fertility decline in response to increased ecological richness should be parameterized by (1) increased age at first reproduction, resulting from slowed development time and higher (extra)somatic investment in maternal human capital (Hill & Kaplan, 1999); (2) optimal (in terms of maternal and infant health and survivorship; between 30 and 41 months) average birth interval because this allows parents to focus time and energy on building somatic, social, and knowledge capital in each individual, high-quality offspring (Kaplan, 1996) without compromising their own health (Schummers et al., 2018); and, possibly, (3) late age at last reproduction because ecological richness may buffer extrinsic threats to survival for both mothers and offspring that are associated with advancing maternal age (Towner et al., 2016).

Economic/investment models focus on the costs and benefits of allocating resources among life-history traits to explain fertility decline, considering the economic and social changes associated with the recent demographic transition. In particular, these models emphasize the rising costs of human capital (especially in the forms of education and skilled maternal work), with such costs increasing as economic sectors become increasingly knowledge-based. Life-history theory suggests that parental fitness, under knowledge-economy conditions, may benefit from accumulating parental human capital and delaying reproduction as long as possible and then focusing resources on generating only perhaps two or three very-high-quality offspring (Becker, 1992; Caldwell, 1982; Kaplan, 1996; Mace, 1998) while continuing to invest in their own somatic resources (healthful longevity with continued knowledge and wealth acquisition; Becker 1992; Budig & England, 2001; Galor, 2011; Low et al., 2002; Turke, 1989). A recent addendum to this approach comes from a case study with Maya speakers from rural Mexico, which indicates that lower fertility in the context of a transition to a market economy is associated with lower economic diversity, lower income, and less wealth accumulation at a household level, but also with higher per capita income for individual household members (Hackman & Kramer, 2021). Nevertheless, the simplest version of this model predicts that fertility declines associated with increasing ecological richness and human capital should be mainly explained by (1) very long reproductive deferral (i.e., very late age at first reproduction) because this allows parents, especially prospective mothers, to establish and cement their own social, economic, and knowledge resources through education prior to beginning reproduction; (2) safe but not necessarily extended interbirth interval (24 months between birth and next conception; World Health Organization 2005:17) because birth-to-conception intervals in the two-year range can reduce the total impact and length of career and savings interruptions for highly educated, working mothers who have only a few children (Ekert-Jaffé et al., 2002); and (3) an age at last reproduction that is one or two safe intervals (i.e., approximately 30 to 82 months) later than age at first reproduction because highly educated working mothers are expected to focus on accruing further economic resources, which can be translated into various kinds of capital for themselves, their partners, and their children (Ekert-Jaffé et al., 2002).

Cultural transmission models propose that the decline in fertility results from socially learned changes in perceptions of the value of having children, ideal family size, and the desirability of effective use of modern, reliable family planning methods. How these perceptions are socially learned may be structured by the density and shape of kin networks and/or by the status and social position of both the norm modeler and the norm follower, among other factors. Generally, these models assume that declining fertility begins with the adoption of fertility-reducing behaviors and norms (i.e., contraceptive use and/or reproductive deferral) by culturally influential, high-social-status people. These norms—which may or may not be fitness-enhancing to the most affluent members of a population (Stulp & Barrett, 2016)—are then transmitted to and then among other, less-affluent groups via social networks, mass media, and other institutions (Basu, 1993; Kaplan et al., 2003; Schuler & Hashemi, 1994). These so-called prestige biases presumably evolved because they offer useful heuristics that, on average, are fitness-enhancing but can be fitness-costly in the cases of some particular behaviors (Boyd & Richerson, 1988; Colleran, 2016; Richerson & Boyd, 2005). Such cultural models can and should allow for the fact that people from the lowest socioeconomic position are likely to face major sociocultural, political, and financial barriers—as well as kin influences—to adopt behaviors they might otherwise emulate and share (Kramer et al., 2021). There may also be nearly a full generation time lag between availability of reliable contraception and widespread norm changes (Kramer et al., 2021). An example can be seen in rural Poland, where market integration associates with less-dense kin networks, which appears to enhance the transmission of valuing relatively low fertility (Colleran, 2020). We interpret this class of models as predicting a number of patterns. (1) Given that most cultural transmission models are focused on prestige bias, when looking at cross-sectional data we expect late ages at first reproduction and long birth intervals in the highest socioeconomic position. (2) Cultural transmission models predict, cross-sectionally, almost indistinguishable life-history/fertility behaviors between the highest and next highest socioeconomic status groups (i.e., those groups who have an impulse to copy the high status ones and who are also not prevented financially or socially from doing so). (3) People in the lowest socioeconomic positions will experience only very slight fertility declines, at least within the first generation of demographic shift. Lastly, there should be (qualitative) evidence from the cultural environment that having three or fewer children is socially or psychologically desirable.

We underscore here that the three classes of models all reflect combinations of patterns observed in various transitioning human populations. Further, we acknowledge that both use of heuristics to learn behaviors from other members of a population (Colleran, 2016; Hackman & Kramer, 2021; Kramer et al., 2021) and management of wealth, knowledge, and other forms of socioeconomic capital in a given environmental context, i.e., one characterized by more or less extrinsic risk, are core aspects of life-history tactics and evolved human biology (Allen & Nettle, 2019; Pepper & Nettle, 2017).

## Study Justification and Aims

Numerous studies have investigated the extent to which and the ways in which fertility and its related life-history variables (e.g., age at first and last reproduction, interbirth interval, and birthing density) vary with markers of socioeconomic position to understand human reproductive trade-offs immediately before or immediately after the most recent demographic transition (e.g., Colleran et al., 2015; Dribe et al., 2014; Goodman et al., 2012; Griskevicius et al., 2011; Hill & Kaplan, 1999; Lawson et al., 2012; Mace, 2014). In general, this evidence suggests that, in pre–demographic transition populations, greater access to resources implies greater numbers of offspring and higher fitness, such that individuals with higher social status also have higher fertility within their groups (Borgerhoff Mulder & Beheim, 2011; Clark & Hamilton, 2006; Cronk, 1991; Cummins, 2009; Hill & Hurtado, 1996; von Rueden et al., 2008). However, in post–demographic transition populations, this relationship between social hierarchy and fitness is complicated and difficult to interpret, with most cross-sectional analyses generally suggesting that fertility declines with increased access to material resources, but with the majority of high-quality, longitudinal analyses indicating a likely positive association between income and fertility (Shenk et al., 2016; Stulp & Barrett, 2016). Additionally, the complex relationships between fertility and socioeconomic position during a demographic transition might reflect fitness optimization in terms of extrinsic mortality risks or multigenerational wealth accumulation, but they also potentially reflect behavioral maladaptations (Goodman et al., 2012; Morita, 2018).

With the important exception of recent studies with Maya-speakers from rural Mexico by Kramer and colleagues mentioned above (see Hackman & Kramer 2021; Kramer et al., 2021), there appears to be a gap in our understanding of what fertility dynamics look like right in the crux of transition, i.e., for births occurring as ecological abundances/access to resources increase sharply. That is, while many studies have documented coarse markers of fertility (i.e., crude birth and fertility rates) in transitioning contexts (e.g., Bongaarts 2001; Bongaarts & Watkins, 1996), including in Latin American (e.g., Casterline & Mendoza 2009; Lima et al., 2018) and even the specific Chilean case (e.g., Sanhueza 2007; Szot, 2003), few have investigated the timing of fertility decisions over women’s reproductive careers, and even fewer have taken a life-history-theory-based approach. Additionally, relatively few evolutionary studies have been carried out in low- or declining-fertility contexts regarding age at last reproduction and/or interbirth interval (Knodel, 1982; Towner et al., 2016; Zakharov & Ivanova, 1996; but see Mattison et al., 2018 and Kramer et al., 2021 for important exceptions).

With these gaps in mind, the Chilean case represents an excellent population for study. Chile’s 2018 crude birth rate of 12 per 1,000 and crude death rate of 5.7 per 1,000 individuals are similar to the rates in most of the Global North and lower than in most of the Global South (Instituto Nacional de Estadísticas, 2018). At the beginning of the twentieth century, Chile was clearly pre-transition, with crude birth rates around 40 live births for each 1,000 inhabitants and with death rates fluctuating around 33 per 1,000 (Cerda, 2008). Mortality declined sharply through the middle of the century, reaching a crude mortality rate of 12 per 1,000 by 1960, with much of this decline accounted for by a halving of child mortality from 342 to 1,000 children under 5 years of age ca. 1900 to 120 per 1,000 children under age 5 by 1960 (Kaempffer & Medina, 2006; Villalobos, 2014), and additional declines down to 9 per 1,000 by 2000 (Szot, 2003). Meanwhile, crude birth rates remained as high as 36.3 per 1,000 until ca. 1960 (Szot, 2003). From then on, crude birth rates fell irregularly between 1960 and 1970 and then fell steadily after 1970, reaching 17 per 1,000 by the turn of the century (Cerda, 2008; Villalobos, 2014). This late-twentieth-century phase of steady fertility decline appears to have been driven largely by increases in average birth spacing from around two years to around three years because age at first birth remained constant at ~ 24 years, at least through the 1970s (Sanhueza, 2007). In summary, Chile recently (between 1960 and 2001) underwent a demographic transition. This 40-year horizon for a major demographic transition reflects an accelerated pace of transition relative to the century-long time spans for transitions undergone in the Global North/West.

We note that the late-twentieth-century decline in crude birth rates in Chile maps onto strong, state-sponsored investment in family planning policies and introduction of modern contraception (Sanhueza, 2007). We view widespread adoption of modern contraception as a more parsimonious explanation for the steep, rapid fertility decline than assuming that prospective parents are assessing, consciously or unconsciously, child mortality rates and adjusting birth rates accordingly through other behavioral or social means.

This study aims to analyze variations of fertility and fertility-linked life-history traits in relation to socioeconomic indicators during the height of the accelerated demographic transition in Chile using life-course and life-history-theory-based perspectives. Specifically, we seek to examine lifetime fertility, the timing of age at first and last reproduction, average interbirth intervals, birthing density, and their relationship with socioeconomic markers for Chilean women born between 1963 and 1967. The approach of this study is to describe selected life-history traits to reflect on how individuals may differ in their reproductive behaviors resulting in lowered fertility, depending on their socioeconomic position in Chilean society.

## Methods

### Population of Study and Ethics

Our analyses focus on sociodemographic and reproductive history data from a single-round survey, carried out in 2013, from a cohort of 6,802 Chilean women living in nuclear family households and who were born between 1961 and 1970 (aged 43–52 years at the time of data collection). Nuclear households were used to reduce the analytical complexity involved in accounting for multiple forms of household composition. We restricted our analyses to women who had completed their reproductive careers because we sought to characterize fertility and reproductive timing across whole reproductive lifespans, including age at last birth, which tends to be under-studied from an evolutionary life-history angle (Towner et al., 2016). We also wanted to ensure that we captured last interbirth intervals, which are often longer and feature higher levels of investment and more careful planning than earlier ones (Kramer et al., 2021; McKerracher et al., 2017). The women in the particular cohort that we selected had reproductive lifespans that align with the time period we thought to be of interest behaviorally, i.e., the crux of the fertility decline in the Chilean demographic transition (Szot, 2003). Moreover, the age range of the cohort was based on the values of age at menopause in Chilean women (Mean = 47.5 years, SD = 4.3 years; Blümel et al., 2000) to ensure that our assumption of selecting individuals with completed reproductive careers was appropriate for the population of study.

The data were derived from the *Encuesta de Caracterización Socioeconómica Nacional* (CASEN) (Ministerio de Desarrollo Social, 2014). This database, from a single-round survey, contains individual-level information concerning household composition, health, and socioeconomic indicators publically available for research and public policy development from the Ministry of Social Development of the Chilean government.

As the database is of public access, it does not contain identifying information for individual respondents and ensures their anonymity, in agreement with standard considerations regarding research ethics (Kitchin, 2013; Mittelstadt & Floridi, 2016; Zwitter, 2014).

### Characterizing Socioeconomic Position

Socioeconomic position (hereafter, SEP) as of 2013 was defined with variables related to education, income, health, housing, property ownership, and living conditions (Table 1). These variables were selected because they were available in the CASEN database, and because previous work identified them as the most reliable indicators of socioeconomic position (Atria, 2005; Harvey, 2008; Krieger, 2001; Lynch & Kaplan, 2000; Teevan, 1985; Winkleby et al., 1992).

**Table 1:** Variables included in the factor analysis of mixed data used to define socioeconomic position (SEP). Years of schooling refers to the number of years in the educational system (e.g., 12 years), while educational attainment level refers to the stage the individual has attained (e.g., completed high school). Autonomous income refers to all kinds of income obtained by all members of the household, while labor income refers only to income produced via paid labor. Variables related to health, pension, housing, property ownership, and living conditions are discrete variables involving more than two categories

Variable	Description	Category
ESC	Years of schooling	Education
Educ	Educational attainment level	Education
ytrabajoCorh	Labor income of household	Income
yautcorh	Autonomous income of household	Income
ysubh	State subsidy income of household	Income
ytotcorh	Total income of the household	Income
ypchtot	Per capita income of the household	Income
s14	Healthcare insurance system	Health
o29	Pension system	Pension
r19	Mobile phone ownership	Material ownership
v1	Type of housing	Housing
v2	Wall material	Housing
v4	Floor material	Housing
v6	Roof material	Housing
v9	Ownership of the dwelling	Property ownership
v11	House square meters	Housing
v12	Ownership of the household	Property ownership
v23	Water source system	Living conditions
v24	Water distribution system	Living conditions
v25	Sewerage system	Living conditions
v26	Electricity system	Living conditions

A factor analysis explained 9.959% of the variability in the sum of dimensions 1 (6.575%) and 2 (3.384%). The first dimension was related mainly to income, education, and health variables, whereas the second was related mainly to housing and living conditions. Only the first dimension was used to characterize SEP because it loaded predominantly onto the variables most commonly used to measure SEP and to assess health and reproductive inequities, such as income and education. The coordinates of dimension 1 were scaled to produce a single, continuous SEP variable (Booysen et al., 2008), wherein individuals of lower SEP have lower scores and those of higher SEP have higher scores. The factor analyses of mixed data were carried out using the *FactoMineR* package (Le et al., 2008) in the statistical environment *R* (version 4.0.3; R Core Team, 2020).

Because geographic region, ethnic self-identification, and urban versus rural living are likely to structure the observations of both life-history trait indicators and the SEP indicators, they are unlikely to be statistically independent, violating a main assumption of linear regression modeling. To account for this, we clustered the data by region, municipality, ethnic self-identification, and urban versus rural living, adding random slope terms for each of these variables.

### Life-History Traits

All life-history traits—number of offspring, age at first reproduction, age at last reproduction, average interbirth interval, and number of births per reproductive lifespan year (hereafter, birthing density)—directly reflect or are derived from the self-reports of individual women represented in the CASEN database. Whereas number of offspring data pertain to all women in the cohort (*n* = 6,802), age at first and last reproduction pertain only to those women who reported having given birth to at least one child (*n* = 6,371), and the average interbirth interval and birthing density data pertain to the smaller subset of these women who reported two or more children (*n* = 6,061).

Number of offspring refers to the number of children ever born to each woman. Age at first reproduction is the age in years at which a woman reported giving birth to her first child, marking the beginning of her reproductive lifespan. Age at last reproduction was calculated by subtracting the age in years of the youngest child reported to have been born to the mother from the mother’s own age in years, at the time of interview.

Average interbirth interval and birthing density are life-history traits that are meant to characterize the reproductive scheduling of individuals. The average interbirth interval does so by focusing on the timing of reproduction, whereas birthing density measures a fertility outcome. In this study, average interbirth interval represents the average time elapsed between successive births, calculated by subtracting a respondent’s age at first reproduction from her age at last reproduction and then dividing the resulting reproductive lifespan (in years) by the woman’s total number of offspring. Birthing density denotes the number of offspring per reproductive year. This variable was calculated by dividing the total number of offspring of an individual by the length of her reproductive lifespan (i.e., subtraction of age at last reproduction and first reproduction).

### Statistical Analyses

Distribution-specific generalized linear regression models were used to analyze the relationship of SEP, region, municipality, rural/urban area, and ethnic self-identification (i.e., predictor variables) on each life-history trait (i.e., response variable). A model selection procedure—Akaike’s Information Criterion (AIC)—was used to select the best model from a pool of simple, multiple, and stepwise AIC-based distribution-specific generalized linear regression models as well as mixed-effect versions of each of these models. For the mixed-effect models, we added random slope terms for region, rural/urban area, and ethnic self-identification.

Since the distribution of the residuals for both age at first reproduction and birthing density were heteroscedastic when regressed on SEP, we log-transformed these variables to meet regression assumptions. SEP was square-root-transformed in the model for number of offspring to meet regression assumptions. A Poisson regression model was fitted for number of offspring because no overdispersion was detected (*c* = − 0.344, *p* = 1).

The analyses were done in the statistical environment R. The *lme4* R package was used to fit the mixed-effect models (Bates et al., 2015). Overdispersion was evaluated with the *dispersiontest* function of the *AER* R package (Kleiber et al., 2020).

## Results

### Average Interbirth Intervals and Birthing Density

**Fig. 1:**
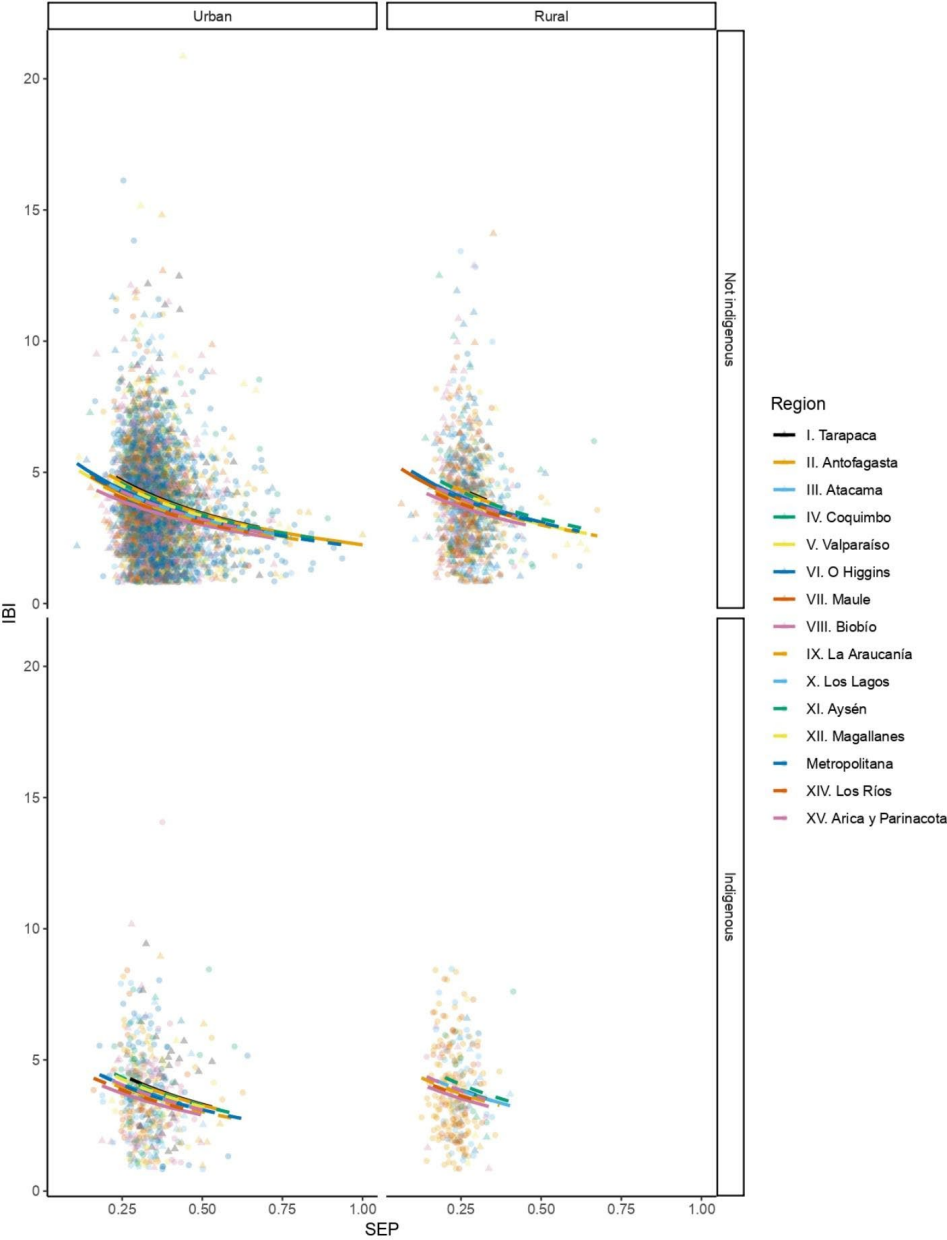
Relationship between socioeconomic position (SEP) and average interbirth interval (IBI), grouped by ethnic self-identification, geographical region, and urban-rural living. There is a line and a point shape for each geographical region. The columns show the relationship in urban (left) and rural (right) settlements. The rows show the relationship among individuals that self-identify (or not) as Indigenous

Birth spacing ranges from 1 year to 21 years (Table 2). The mean (SD) average interbirth interval for the full cohort is 3.77 (± 1.872) years, with a median of 3.5 and a mode of 3 (Table 2); these central tendencies vary by decile, with the mean, median, and modal average interbirth intervals in the highest SEP decile being 3.11, 3, and 2; 3.9, 3.667, and 3 in the fifth SEP decile; and 4.057, 4, and 3 in the lowest. Based on the model selection process, a multiple Gamma regression model was selected, with SEP, geographical region, ethnicity, and rural-urban variables as predictor variables. The model shows that, as SEP increases, the average interbirth intervals becomes shorter (coeff. (SD ± 0.025) = 0.307, *p* < 0.000), while geographical region, ethnicity, and rural-urban living are kept constant (Table 3; Fig. 1).

**Table 2:** Summary statistics of number of offspring, age at first and last reproduction, average interbirth interval, and birthing density

Life-history trait	Sample size	Mean	Median	Mode	SD	Min	Max
Number of offspring	6,802	2.665	3	2	1.361	0	13
Age at first reproduction	6,371	22.54	22	20	5.06	10	47
Age at last reproduction	6,371	32.97	33	32	5.309	15	49
Average interbirth interval	6,061	3.77	3.5	3	1.872	1	21
Birthing density	6,061	0.35	0.286	0.333	0.213	0.048	1

**Table 3:** Summary table of the regression models used to analyze the relationship of socioeconomic position (SEP) with average interbirth intervals (BI), birthing density (BD), age at first reproduction (AFR), age at last reproduction (ALR), and number of offspring (N° Off.). A Poisson regression model is reported for number of offspring

	IBI (*n* = 6,061)			BD (*n* = 6,061)			AFR (*n* = 6,371)			ALR (*n* = 6,371)			Nº Off. (*n* = 6,802)		
	Coeff	SD	*p*	Coeff	SD	*p*	Coeff	SD	*p*	Coeff	SD	*p*	Coeff	SD	*p*
SEP	0.307	0.025	< 0.000	1.174	0.087	< 0.000	0.698	0.031	< 0.000	−0.82	0.754	0.277	−1.338	0.11	< 0.000
I. Tarapacá	0.137	0.012	< 0.000	−1.69	0.048	< 0.000	2.811	0.018	< 0.000	–	–	–	1.907	0.076	< 0.000
II. Antofagasta	0.003	0.013	0.814	−0.003	0.051	0.946	-0.001	0.02	0.959	–	–	–	−0.074	0.056	0.181
III. Atacama	0.019	0.012	0.105	0.056	0.048	0.243	0.001	0.019	0.96	–	–	–	−0.033	0.051	0.525
IV. Coquimbo	0.018	0.011	0.107	0.067	0.047	0.147	0.035	0.018	0.053	–	–	–	−0.144	0.051	< 0.01
V. Valparaíso	0.025	0.01	< 0.05	0.089	0.041	0.032	0.043	0.016	< 0.01	–	–	–	−0.196	0.045	< 0.000
VI. O’Higgins	0.017	0.01	0.096	0.066	0.042	0.119	0.049	0.016	< 0.01	–	–	–	−0.215	0.046	< 0.000
VII. Maule	0.023	0.01	< 0.05	0.076	0.043	0.074	0.059	0.017	< 0.001	–	–	–	−0.198	0.046	< 0.000
VIII. Bío-Bío	0.043	0.009	< 0.000	0.169	0.039	< 0.000	0.077	0.015	< 0.000	–	–	–	−0.212	0.042	< 0.000
IX. Araucanía	0.028	0.01	< 0.01	0.098	0.042	0.019	0.069	0.016	< 0.000	–	–	–	−0.133	0.045	< 0.01
X. Los Lagos	0.02	0.01	0.06	0.077	0.044	0.077	0.045	0.017	< 0.01	–	–	–	−0.182	0.047	< 0.001
XI. Aysén	0.005	0.012	0.672	−0.017	0.041	0.739	0.012	0.02	0.549	–	–	–	−0.11	0.056	0.049
XII. Magallanes	0.008	0.013	0.48	0.049	0.042	0.347	0.051	0.02	0.01	–	–	–	−0.245	0.058	< 0.000
XIII. Metropolitana	0.02	0.009	0.022	0.72	0.038	0.057	0.048	0.015	< 0.01	–	–	–	−0.157	0.04	< 0.001
XIV.Los Ríos	0.034	0.011	< 0.01	0.12	0.045	< 0.01	0.055	0.017	< 0.01	–	–	–	−0.175	0.049	< 0.001
XV. Arica y Parinacota	0.021	0.012	0.091	0.07	0.05	0.159	0.025	0.019	0.197	–	–	–	−0.105	0.054	0.051
Urban	0.137	0.012	< 0.000	−1.69	0.0486	< 0.000	–	–	–	–	–	–	–	–	–
Rural	0.015	0.004	0.001	0.053	0.019	< 0.01	–	–	–	–	–	–	–	–	–
Not Indigenous	0.137	0.012	< 0.000	−1.69	0.0486	< 0.000	–	–	–	–	–	–	–	–	–
Indigenous	0.012	0.006	0.031	0.038	0.022	0.093	–	–	–	–	–	–	–	–	–
R^2^	–			0.032			0.078			< 0.000			–		
AIC	23595.47			9276.576			−1993.4595			39356.22			23510.24		

**Fig. 2:**
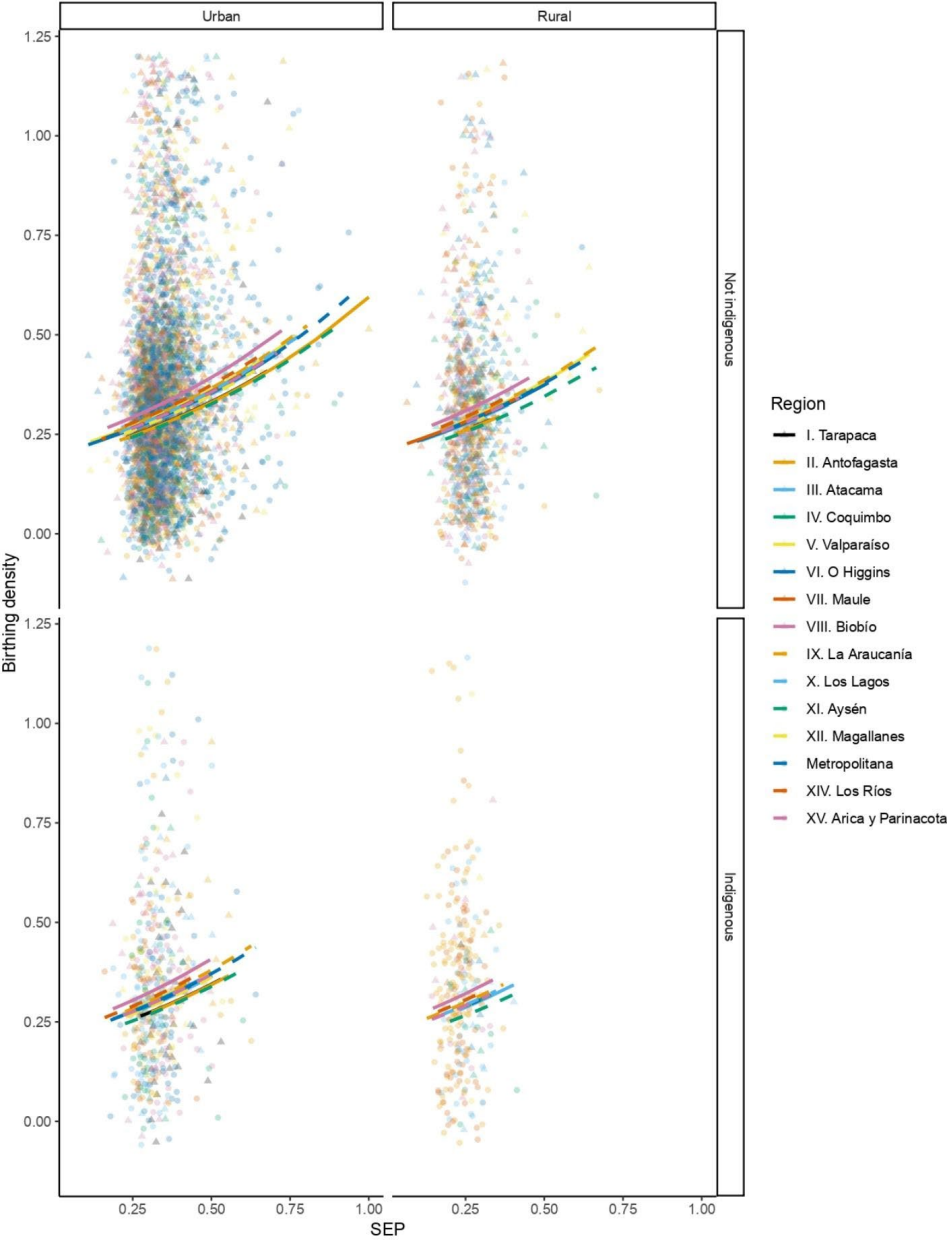
Relationship between socioeconomic position (SEP) and birthing density, grouped by ethnic self-identification, geographical region and urban-rural living. There is a line and a point shape for each geographical region. The columns show the relationship in urban (left) and rural (right) settlements. The rows show the relationship among individuals that self-identify (or not) as Indigenous

Mean (SD) birthing density is 0.35 (± 0.213), with a median of 0.286 and a mode of 0.333 offspring per reproductive year (Table 2). Birthing density ranges from its least dense or most diffuse at 0.048 offspring per reproductive year to its most dense at 1 offspring per reproductive year (Table 2). Central tendencies of birthing density vary by SEP decile, with women from the highest decile, the fifth decile, and the lowest decile giving birth to, respectively, a mean of 0.0.427, 0.324, and 0.315, a median of 0.333, 0.273, and 0.25, and a mode of 0.5, 0.3, and 0.25 children per reproductive span year. A multiple linear regression model was fitted to observations pertaining to the relationship between socioeconomic position, geographical region, ethnicity, and rural-urban living with birthing density, based on the results of model selection. This model shows that birthing density increases exponentially by 3.234 offspring per reproductive year with each increase of one unit of SEP (coeff. (SD ± 0.087) = 1.174, *p* < 0.000), when geographical region, ethnicity, and rural-urban living remain constant (Table 3; Fig. 2). These results suggest that individuals of higher SEP compress their reproduction temporally in comparison to those of lower SEP.

### Age at First and Last Reproduction

**Fig. 3:**
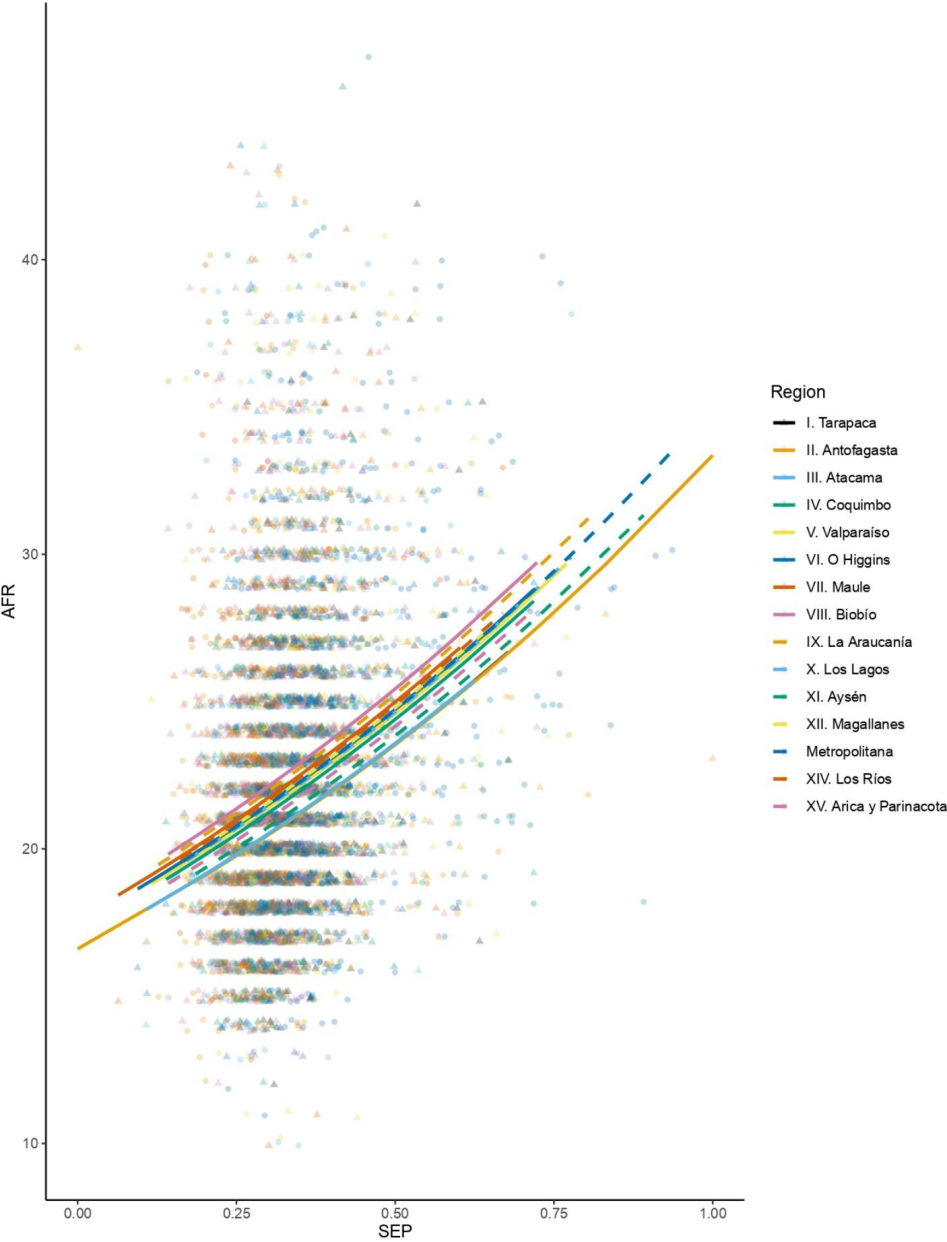
Relationship between socioeconomic position (SEP) and age at first reproduction (AFR), grouped by geographical region. Each geographical region is indicated by a distinctive point and line color

**Fig. 4:**
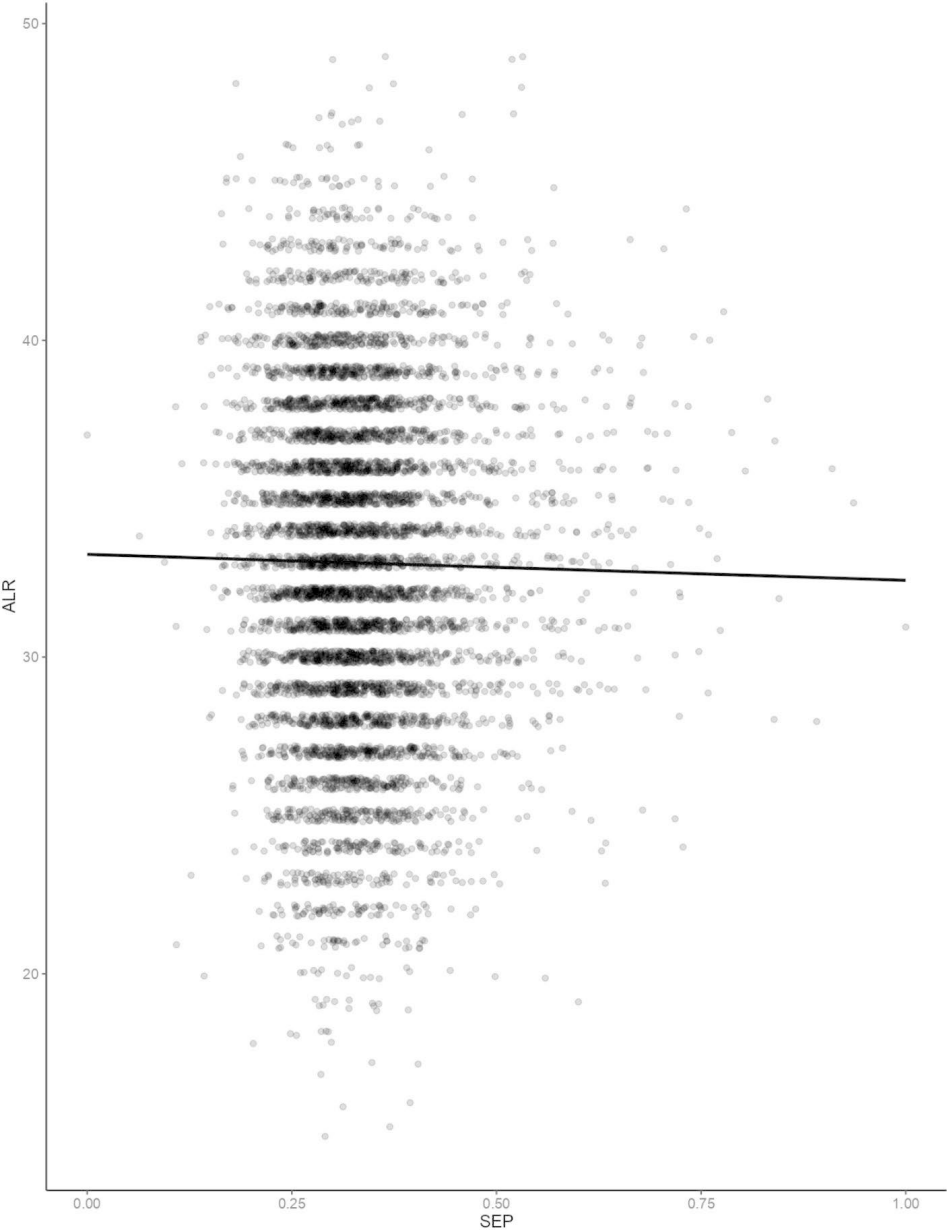
Relationship between socioeconomic position (SEP) and age at last reproduction (ALR)

Mean (SD) age at first reproduction in the cohort is 22.54 (± 5.06) years old, with a median of 22 and a mode of 20 (Table 2). The earliest reported first birth in the cohort was to a 10-year-old; the latest was to a 47-year-old (Table 2). A multiple linear regression model was selected with SEP and geographical region as fixed effects. The model shows that, with each single-unit increase in SEP, women exponentially delay their reproductive onset by 2.009 years (coeff. (SD ± 0.031) = 0.698, *p* < 0.000), holding geographical region constant (Table 3; Fig. 3). Mean (SD) age at last reproduction is 32.97 years old (± 5.309), with a median of 33 and a mode of 32; age at last reproduction ranges from 15 years to 49 years (Table 2). A linear regression model was selected and fitted to the data pertaining to the relationship between SEP and age at last reproduction, and this model shows that age at last reproduction decreases by 0.82 years with an increase of one unit of SEP (coeff. (SD ± 0.754) = − 0.82, *p* = 0.277) (Table 3; Fig. 4). These results suggest that individuals of higher SEP generally have shorter reproductive lifespans due to both delayed reproductive onset and early reproductive cessation in comparison to those of lower SEP.

### Number of Offspring

**Fig. 5:**
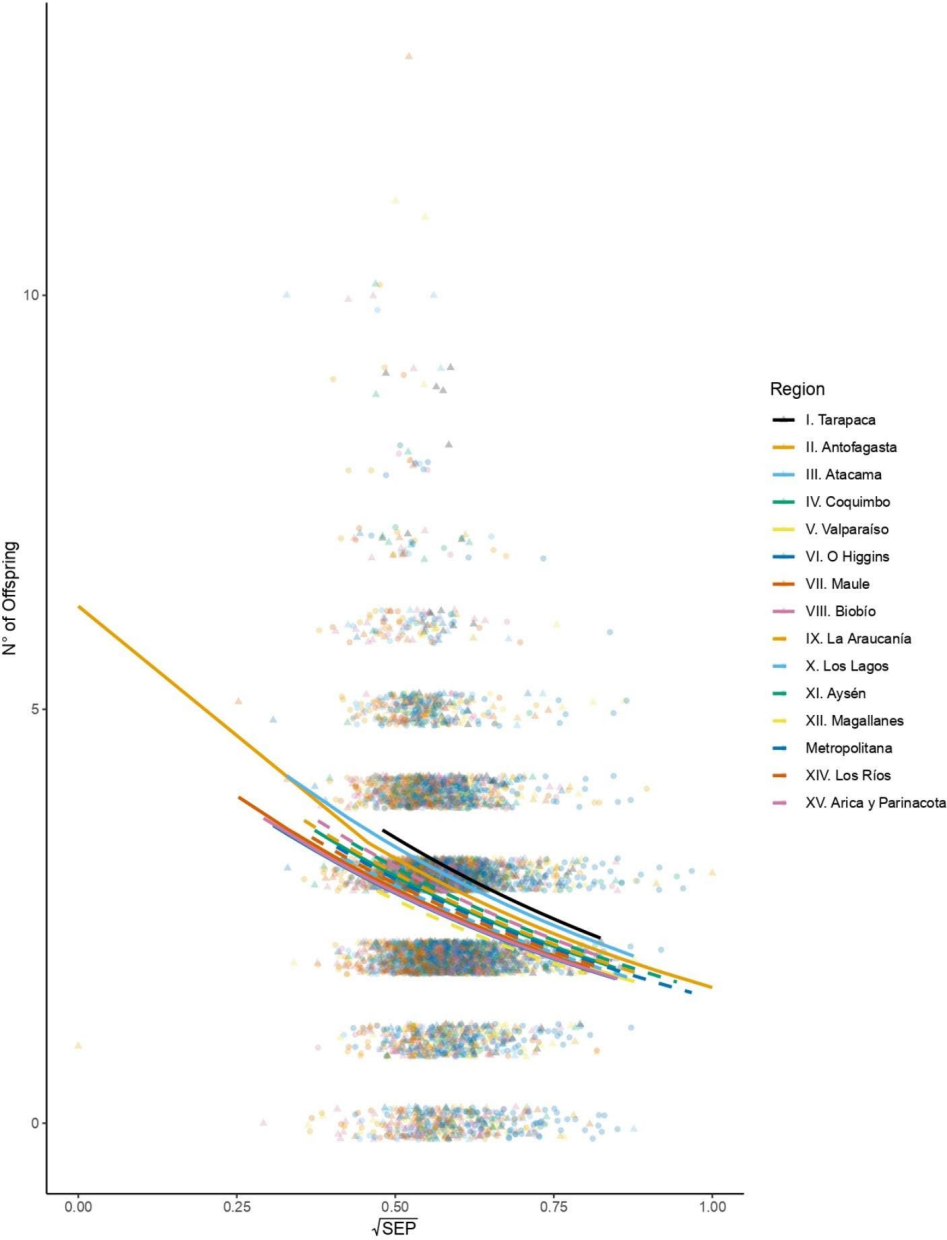
Relationship between socioeconomic position (SEP) and number of offspring, grouped by geographical region. There is a line and a point shape for each geographical region. The socioeconomic position (SEP) is square-root transformed

The average (SD) number of offspring born to each mother within the cohort was 2.665 (± 1.361), with a median of 3 and a mode of 2 (Table 2). The minimum reproductive outcome was 0, and the maximum was 13 (Table 2). A Poisson multiple regression model was selected and fitted to analyze the relationship between socioeconomic position, geographical region, and number of offspring. The model shows that the number of offspring exponentially decreases by 1.791 children for every unit of increase in SEP (coeff. (SD ± 0.11) = − 1.338, p < 0.000) while holding geographical region constant (Table 3; Fig. 5). This result suggests that individuals of higher SEP reduce their lifetime fertility in comparison to those of lower SEP.

## Discussion and Conclusion

### Summary of Main Findings

Using cross-sectional data from a cohort of Chilean women who reached reproductive maturity in the middle of Chile’s most recent demographic transition, this study examined variability in fertility and life-history tactics in relation to variability in availability and distribution of socioeconomic resources. We found that lifetime fertility decreases steadily with socioeconomic position (SEP). We further showed that, at the level of life-history tactics, age at first reproduction and birthing density increase, whereas both age at last reproduction and average interbirth interval decrease as SEP increases. In particular, the highest SEP women in the sample generally tended to delay beginning their reproductive careers into their early thirties and then packed births densely into a relatively short time span, mostly by age 37. Furthermore, women from the highest socioeconomic stratum seem to be distinct from all other strata with respect to their shorter average interbirth intervals and high birthing density. We infer from this detectable gap between the women of highest SEP and those of the next tiers of SEP that women from the middle strata were unlikely to be closely emulating the reproductive behaviors or life-history tactics of the most affluent women, at least not within the cohort we examined (cf. Colleran et al., 2015).

In light of the predictions we derived from the three classes of semi-complementary socioecological models put forward to account for the most recent demographic transition globally (Shenk et al., 2013), these findings may align with those of all three, perhaps slightly favoring the investment-focused perspectives. To reiterate, all classes of models predict reduced lifetime fertility and delayed age at first reproduction as resource abundance increases, and all are compatible with a shortened reproductive span. The only possibly distinguishing feature of the data with respect to the models concerns the relatively short average interbirth intervals and high birthing density of women of very high SEP, which, notably, are near health-and-fitness optima for well-nourished and well-supported women. This pattern implies that women in this cohort with very high levels of education and income stop reproducing relatively early and then may reallocate their time and energy through their late thirties and their forties to things other than reproduction, perhaps investing in themselves or in their household’s social, economic, and/or political assets (cf. Kaplan, 1996).

A corollary of these inferences is that the trade-offs between offspring quality and quantity in this cohort are not straightforward. The potential effects of local history and cultural transmission and adoption of fertility norms are also not easy to identify. Rather, the picture is complex, likely involving trade-offs within and among multiple socioecological spheres, both embodied and not (Kaplan et al., 2003; see also evidence from a Polish case by Colleran et al., 2015, and across a variety of cultural and historical contexts reviewed by Lawson & Borgerhoff Mulder 2016). In what follows, we unpack some of these complex trade-offs when thinking through the Chilean fertility and life-history data in relation to previous work on other comparable populations undergoing fertility transitions. We also discuss some of the implications of our findings with respect to both health and evolutionary fitness.

### Comparison to Transitions in Contexts Outside the Global North/West

In the broadest sense, our findings are consistent with previous research investigating the relationship between fertility, resource abundance, and SEP. As with most previous cross-sectional studies on transitioning or post-transition human populations (e.g., Bongaarts 2001; Bongaarts & Watkins, 1996; Itaboraí, 2015; Lawson & Borgerhoff Mulder, 2016; Shenk et al., 2013), lifetime fertility in this Chilean cohort is negatively associated with SEP. Additionally, our results accord with previous studies on the timing of age at first reproduction in relation to SEP and other indicators of resource abundance and access in Latin America. This body of work generally indicates that first reproductions tend to occur early (in adolescence) in lower socioeconomic strata of transitioning Latin American populations (e.g., Casterline & Mendoza 2009; Hackman & Kramer, 2021; Itaboraí, 2015; Kramer & Greaves, 2017; Kramer et al., 2021; Lima et al., 2018; McKerracher et al., 2017).

Our findings, however, contribute new texture to the picture in two ways. First, regarding average interbirth interval and birthing density, women of higher SEP exert relatively strong control over their reproduction. This autonomy, which is likely enabled in part by increased access to multiple reliable and safe forms of contraception (Perusse, 1993) and which may be enabled in part through extended and well-supported lactation, is expected to favor birth intervals of approximately three to four years, which are associated with relatively low morbidity and mortality risks for both mothers and babies (Blurton Jones, 1986; Palloni & Millman, 1986; Winikoff, 1983). This 3- to 4-year birth spacing strategy, combined with low total fertility, also allows women of higher SEP to invest their time and energy heavily in each offspring, while also reserving energy to invest in their own health, consolidation of socioeconomic resources, and possibly in future grandchildren, ensuring lineage stability. That said, while perhaps the observed patterns were to be expected, previous data from other contexts have been highly variable, with high SEP sometimes associated with shorter-than-recommended birth intervals and sometimes with quite long ones (e.g., Holowko et al., 2018; Khan et al., 2016).

Secondly, the age at last reproduction data show that women from the highest socioeconomic strata in this cohort tend, on average, to stop giving birth nearly ten years before menopause. A handful of previous demographic transition analyses (e.g., on Swedish cohorts, reviewed in Omran 2005; see also work by Mattison et al., 2018 on Chinese fertility transitions) have shown that age at last reproduction drops to a few years before menopause in high SEP groups mid- and post-transition. However, both Davison & Gurven (2021) and Towner and collaborators (2016) show that stopping reproduction around a decade before menopause is not uncommon across human populations, particularly natural fertility ones. So, our results regarding flexibility and variation in age at last reproduction are not without precedent, but they are fairly novel within the context of investigating human demographic transitions: the majority of previous demographic transition analyses focus only on the timing of age at first reproduction, while ignoring age at last reproduction on the assumption that this variable is likely to coincide roughly with menopause (e.g., Helle et al., 2005; O’Connor et al., 1998; Peccei, 2001; Sievert, 2014; Wood et al., 1994). Therefore, our results provide additional evidence that low fertility can be linked to earlier age at last reproduction (see also Davison & Gurven 2021) and highlight the need for demographic transition researchers to address a research bias against investigating and reporting the timing of reproductive cessation.

Taking the Chilean average interbirth interval, birthing density, and age at last reproduction data at face value and looking at all three of these variables together, these indicators of reproductive behaviors may relate to the specific historical context of the recent Chilean demographic transition. In particular, we note that the accelerated and recent demographic transition in Chile accompanied a violent and rapid installation of a neoliberal economic system under and immediately following the dictatorship of Augusto Pinochet (1973–1990). As a result of the rapid imposition of a neoliberal order, Chilean society has developed high levels of socioeconomic inequity, featuring rampant status competition, restricted social mobility, and limited social welfare programming relative to other states in the region (Contreras & Ffrench-Davis, 2012; Fischer et al., 2003; Torche, 2005a). These striking inequities may have imposed especially high penalties on higher-birth-order births and imbued higher socioeconomic strata with especially strong incentives to consolidate resources among only a few children (Lawson & Borgerhoff Mulder, 2016; Shenk et al., 2016), possibly leading to early reproductive cessation for those women who were in the position to control their fertility through newly available, reliable contraceptive methods (Mattison et al., 2018). We further speculate that, if these sociopolitical forces contributed to delays in average age at first reproduction and early reproductive cessation, close birth spacing and dense reproduction could result from pressures on both ends of the reproductive span. In keeping with this hypothesis, delayed age at first reproduction combined with concerns about health and economic risks associated with older motherhood appear to have driven declines in interbirth interval in affluent segments of at least one other transitioning population (Cebu, Philippines; Upadhyay & Hindin 2005). Future research should explore whether and to what extent other societies that underwent an accelerated demographic transition within a context of sharp rises in inequality and sudden reduction of welfare policies also exhibit earlier-than-expected end of reproductive life-span combined with relatively high birthing density in the segments of the population that have the most power to control their own fertility.

### Implications for Demographic Transition Theory and Contemporary Public Health

The results reported here are consistent with the view that systematic variations in life-history tactics among socioeconomic strata underlie different patterns of fertility decline in the late stages of the recent demographic transitions in Chile and elsewhere (Lawson & Borgerhoff Mulder, 2016). In particular, we observed pronounced differences in lifetime fertility, timing of onset and cessation of births, birth spacing, and birthing density among women from lower, middle, and higher SEP groups. These observations highlight the value of studying life-history traits such as age at last reproduction, average interbirth interval, and birthing density—in relation to fertility (Nepomnaschy et al., 2020). This approach has the potential to generate or elaborate mechanistic hypotheses that might throw context-specific light on the evolutionary puzzle presented by declining fertility in relation to increasing resource abundance. The life-history-focused approach also has implications for public health.

With regard to age at first reproduction, our findings agree with those of the overwhelming majority of previous studies (Dribe et al., 2014; Lesthaeghe, 2010, 2014) in that they suggest that delaying the onset of reproduction is a main mechanism through which fertility declines as resource richness increases. This mechanism warrants the special attention it has received in the literature. Unlike extending birth intervals or ending reproduction early, delaying age at first reproduction does not necessarily require access to reliable methods of contraception—either social (e.g., straightforward paths to marriage dissolution or sexual avoidance) or technological (e.g., intrauterine devices, hormonal contraceptives)—to have profound demographic consequences. Rather, it requires channeling more time and energy resources into a variety of other things in the adolescent and early adulthood stages of the life course, including somatic growth, attractiveness to high-quality mates, and accrual of knowledge (particularly through formal education), skills (possibly through formal education or apprenticeships), and material wealth. These somatic (“embodied”) and extrasomatic resources accrued through adolescence can subsequently be channeled into producing and nurturing relatively high-quality offspring (Hill & Kaplan, 1999; Kaplan, 1996; Kaplan et al., 2000, 2003; Lancaster & Kaplan, 2010; Mace, 1998). This strategy is likely to be fitness-enhancing under conditions in which ecological resources are abundant but unequally distributed (Shenk et al., 2016). Moreover, increased age at first reproduction (and resulting lifetime fertility declines) should be an evolutionarily-expected outcome in a socioeconomic and political environment such as that of the Chilean study cohort.

Our results indicate that age at last reproduction may be an indicator of fertility decline with a level of impact similar to age at first reproduction. Although our results are not unprecedented (Omran, 2005; Mattison et al., 2018; see also Snopkowski et al., 2014; Towner et al., 2016; Wood et al., 1994), they highlight that reproductive cessation can be behaviorally decoupled from the biological constraint of menopause by nearly a decade (Davison & Gurven, 2021; Towner et al., 2016). With this pattern in mind, future demographic transition studies should seek to account for variation in age at last reproduction and dig into the proximate mechanisms through which high SEP women (or couples) end reproduction long before they become biologically incapable of conceiving and supporting additional children. Some obvious candidate explanations include achievement of desired fertility, combined with unfettered access to reliable contraception (Singh et al., 2017); desire or need to reenter the labor market, making use of knowledge and skills acquired through formal education prior to beginning reproduction (Spéder & Kapitány, 2015); familiarity with the evidence that risks of pregnancy, labor, and delivery complications as well as of poor birth outcomes increase with maternal (and paternal) age (Castro Martin, 1995); need to invest energy to ensure older children delay their reproduction successfully (Galbarczyk & Jasienska, 2013; Kaplan, 1996; Lahdenperä et al., 2012); or desire to emulate successful, high-status women in other countries, who tend to end reproduction in their thirties (Watkins & Hodgson, 2019). We note that these are just plausible scenarios, and many other possible conceptual models or scenarios could also fit the data we observed. Irrespective of the reasons women have for ceasing reproduction when they do, paying more attention to age at last reproduction will likely throw light on how women and their families allocate resources as they and their earlier-born children age, facilitating better measurement of expected fitness a few generations down the line.

Our main finding with respect to average interbirth interval and birthing density—that high SEP women pack a relatively small number of births together into a short period of time—may reflect two plausible stories. First, average interbirth intervals of around three years and high birthing density when household-level ecological conditions are abundant may largely be driven by external factors shortening the time window in which to have children from both ends. To achieve desired fertility after social and/or economic consequences of beginning to have children in their thirties or even forties, women in the Chilean cohort may fit their intended two, three, or perhaps four births into a span of only a few years, resulting in fairly short spaces among births. Another plausible story is that many of the women are following 3-year birth spacing strategies that resemble both cross-cultural norms and possibly biological optima (Davison & Gurven, 2021) and then stop reproducing when they have achieved their desired fertility. In this scenario, age at first reproduction, average interbirth interval, birthing density, and access to reliable contraceptive methods interact to drive down age at last reproduction. A variation on this latter possibility is that increased availability of ecological resources supports earlier return to net positive energy balance and earlier resumption of regular ovarian cycling following a birth, increasing the likelihood of conception and closure of the interval (Valeggia & Ellison, 2009). We note that both of these scenarios can potentially be true at the same time insofar as global fertility dynamics appear to reflect some complex combinations of desire-dependent and desire-independent changes (Hruschka et al., 2019).

All three observed patterns in life-history tactics—delayed first reproduction, relatively high birthing density compressed into a few prime reproductive years, and early reproductive cessation in women of higher SEP—have fairly well understood effects on maternal, child, and population health. Delaying age at first reproduction until somatic growth is fully complete generally offers health benefits to both a mother and her children. Fetuses, via the placenta, extract energy and nutrients from maternal bodies (Haig, 1993), meaning that a mother who is still growing will face sharp trade-offs between her own nourishment and the support of the pregnancy. As such, adolescent mothers, especially very young adolescent mothers, are at increased risk for anemia and hypertension and their offspring are at increased risk of perinatal morbidity and death (see data in Kramer & Lancaster 2010; see also World Health Organization 2005). Adolescent mothers also face challenges to advancing in their formal education, which has its own health costs, related to autonomy and empowerment, health literacy, and income. Similarly, risks of chromosomal anomalies, hypertension, fetal growth restriction, and preterm birth increase substantially with older maternal age (Cavazos-Rehg et al., 2015; Lean et al., 2017; Sauer, 2015). Taking these two variables together, then, the trend toward concentrating reproduction in the middle of the reproductive span is likely to generally reduce health challenges faced by Chilean women and children, should all segments of the population be able to access improvements in ecological conditions. However, some of these benefits could be undercut by the trend toward relatively compressed reproduction, if it continues further in the direction it appears to be headed. There is evidence that it takes an average of 24 months for maternal micronutrient profiles to be sufficiently replenished after pregnancy and on-demand lactation, as well as that birth intervals under ~ 30 months are associated with elevated risks of a wide variety of pregnancy and delivery complications (Conde-Agudelo et al., 2005, 2006; DaVanzo et al., 2008; Rutstein, 2005; Schummers et al., 2018). Hence, the World Health Organization recommends waiting at least 24 months after the birth of one child to conceive another, with total birth intervals less than about three years contraindicated, even for well-nourished, well-supported women (WHO, 2005). With these three variables in mind, and after adjusting the WHO recommendations to the Chilean population, public health efforts at reducing health inequalities in Chilean birth outcomes should perhaps prioritize redistributing socioeconomic resources as widely and as equitably as possible; supporting pregnancy prevention/contraception, particularly during both adolescence and perimenopause, for families of lower SEP who would like to lower their fertility; and supporting the maintenance of approximately three-year birth spacing norms in all families, through both policy (e.g., extended or delayed parental leave) and messaging.

### Study Limitations

While our general findings suggest we can gain insight into the recent Chilean demographic transition by examining variations in life-history tactics among women from different socioeconomic position groups, several factors in the study’s design limit its interpretive specificity and power. As such, we identify several cautions. First, the data are cross-sectional rather than longitudinal, meaning that we can only detect patterns of association and not make any strong inferences regarding causation, especially given that the SEP data pertain to 2013 and most of the births occurred between the 1980s and the early 2000s. This data structure also means that we could not derive the strongest predictions with respect to the culture-related class of models, which have an important time-series element. That being said, the cross-sectional approach gives us a snapshot of the patterns involved in a previously understudied context, and our sample is large enough to detect biologically meaningful signals without examining the through-time dynamics. Nevertheless, we plan to carry out additional analyses in future as high quality longitudinal data become available through CASEN, such that we can directly investigate relationships between life-history tactics over the reproductive span and household-level ecological changes.

Second, the CASEN database does not contain information concerning a variety of other life-history traits of potential interest, notably those related to growth, development, and survival/mortality. Also, the CASEN database does not contain information on other socioecological drivers of interest (e.g., climate, population density), which may require harmonization with other databases. This lack of data prevented us from testing the models in ways more directly comparable to those carried out previously (Colleran et al., 2015; Shenk et al., 2013) and from being able to make inferences regarding possible physiological mechanisms for the trade-offs involved (cf. Ellison, 2003).

Lastly, we highlight that the internal explanatory power of the SEP variable was fairly low (9.959%), based on our factor analysis of mixed data, and note again that the SEP data are cross-sectional. Part of the explanation for this low explanatory power may be that conventional measures of SEP and social structure (e.g., standard occupational categories, scales of prestige or social class) (Atria, 2005; Bergman & Joye, 2005) were unavailable in the CASEN database. Additionally, research suggests that SEP measures often show low explanatory power because of the role of social and support networks in reproductive behavior (Colleran, 2020; Newson et al., 2007; Sear & Coall, 2011). Another concern is that the cross-sectional nature of the data means we were unable to assess possible changes through time of household SEP. However, we suspect that changes through time are likely to have had only minor effects on absolute socioeconomic conditions rather than on relative socioeconomic position (and our measures were relative) because social mobility in Chile is very limited (Torche, 2005b). Despite these limitations regarding the measure of SEP employed in this study, we were able to detect the core effects we expected to detect. Moreover, we do not see any reason to think that the approach we took is likely to have biased our results toward any of the three hypotheses, only weakened our power to detect any effect (i.e., we had an across-the-board conservative bias).

### Future Directions

Given that variations in life-history tactics are crucial to understanding how the most recent demographic transition unfolded, and given the ramifications of these variations for health and socioecology, there is a clear need for further research both on the Chilean cohort and on parallel ones in other transitioning populations living in comparable socioecological contexts. In particular, three areas from our analyses, and their limitations, emerged as priorities for further investigation: (1) taking on board a longitudinal perspective within the study cohort, to better understand what is driving variation in average interbirth intervals and birthing density; (2) examining additional cohorts in the CASEN database, but covering a wider historical time span to look at trends through time, ideally in relation to relevant technological and sociopolitical historical particularities (Stulp et al., 2016); and (3) looking more carefully at household-level ecological factors, to the extent that relevant data are available, to identify specific aspects of ecological resource richness that contribute to variations in life-history tactics.

Concerning taking the longitudinal perspective within the cohort, we think it may be possible to discriminate between the two hypotheses we put forward above with respect to whether age at last reproduction constrains average interbirth interval and increased birthing density, or whether average interbirth interval and birthing density norms drive variation in age at last reproduction. Discerning between these possibilities is a necessary step before developing any policy intuitions or recommendations about how to construct and where to target messaging about safe birth spacing.

The second direction—looking at additional cohorts within the database—will allow us to better assess whether the cross-sectional associations we observed reflect a set of wider demographic trends, or an anomalous feature of a crux-of-the-transition cohort. More importantly, taking a longer view could facilitate a time series approach, in which we might be able to link changes in life-history tactics to specific features of history. In particular, it would be interesting to understand the impact of sociopolitical transformations, such as the installation of the neoliberal Pinochet dictatorship, on life-history tactics. Additionally, there is good reason to think that technological drivers related to both fertility management and maternal health could drive acceleration or deceleration of overall life-history strategies (Perusse, 1993; Stearns et al., 2010). And, in keeping with the results of other studies (Shenk et al., 2016; Stulp & Barrett, 2016), rates of inequality and resulting status competition appear to exert their own effects on life-history tactics and reproductive behaviors, independent of ecological abundance. So, we think it will be worthwhile to examine changes in Chilean fertility through time in relation to historical changes in inequality and competitiveness.

Lastly, we plan to extract as much information as possible from the CASEN database to create a more detailed picture of each household’s microecology. Specifically, a growing body of work has highlighted the centrality of social support networks, social transmission networks, cooperative breeding structures, and women’s non–domestic labor participation in driving human life-history tactics and fertility outcomes (Colleran, 2020; Newson et al., 2007; Rindfuss et al., 2003; Sear & Coall, 2011). Testing whether variation in life-history traits associates with variation in coarse proxies for these social organizational factors (e.g., household composition size, presence of babysitting-age older siblings in households) will help untangle how women navigate their fertility under rapidly and dramatically changing conditions.

## Conclusions

In sum, reproductive behavior differs systematically among Chilean women of different SEPs, such that fertility is lowest and most densely packed in the highest SEP groups. We infer that fertility declined in this cohort through a combination of slowed life-history tactics at the beginning of women’s reproductive careers and accelerated reproduction thereafter as ecological-richness increased. These results imply that Chilean women may follow different paths toward fertility declines according to their families’ SEPs. Thus, multiple, interrelated life-history trade-offs may explain the observed variations in reproductive behavior. This set of findings calls attention to the need to go deeper into the multiple life-history tactics underlying observed reproductive behavior. An obvious way forward is to compare these results with previous and subsequent cohorts in Chile, to better characterize the decline in fertility and mortality across the full span of the Chilean demographic transition. Systematic comparisons with cohorts from other rapidly transitioning populations from the Global South might shed further light on how and why more affluent Chilean women shortened their reproductive careers so dramatically.

These findings also raise a number of bigger questions, which we challenge readers to contemplate: How, from an evolutionary perspective, might we make sense of the influence of sociopolitical processes during the demographic transition? What roles do inequality and status competition play in driving differences in life-history tactics? And, to what extent can or should we bring these kinds of questions to bear on real-world phenomena, with their consequences for women and other birthing people, children, and families?

## Data Availability

CASEN database can be accessed in the link http://observatorio.ministeriodesarrollosocial.gob.cl/storage/docs/casen/2013/CASEN_2013_MN_B_Principal_spss.rar.

## Code Availability

The code for the data management and analyses can be found in the link https://github.com/pjve90/RB_SES_CL.
